# Magnetic bead purification of enveloped alphavirus and flavivirus

**DOI:** 10.1016/j.mex.2021.101549

**Published:** 2021-10-14

**Authors:** Bianca Galasso, Shreya Sharma, Barbara Knollmann-Ritschel, Anuj Sharma

**Affiliations:** Department of Pathology, Uniformed Services University of the Health Sciences, Bethesda, MD 20814, USA

**Keywords:** Virus purification, Flavivirus, Alphavirus, Zika virus, Mayaro virus, Magnetic bead purification, Virus purity, Virus titer, Vaccines, Virology

## Abstract

Virus production is an essential part of virology. The quality of virus preparation, in terms of purification and yield, can affect the outcome of experiments. Purified virus is critical for assays, as contamination from cell culture supernatants may affect results. Purified virus is also needed for vaccine preparations. The traditional process of gradient purification of virus is time consuming, with multiple steps spanning over days, and produces significant amounts of biohazard waste. Developing a more efficient alternative that provides a purified virus product is highly desirable. In this study, we performed magnetic bead purification of Zika virus (ZIKV) and Mayaro virus (MAYV) with the Mag4C LV kit from OZ Biosciences. Currently, this kit is only indicated for use with lentiviruses and retroviruses. We found that we were able to successfully concentrate and purify both ZIKV and MAYV using the kit. Therefore, we concluded that the Mag4C LV kit provides a quick and simple alternative to traditional virus purification methods.

• Our protocol is customized by using an alphavirus (MAYV) and flavivirus (ZIKV). This method has not been previously used for these viruses.

## Graphical abstract

**Figure:**
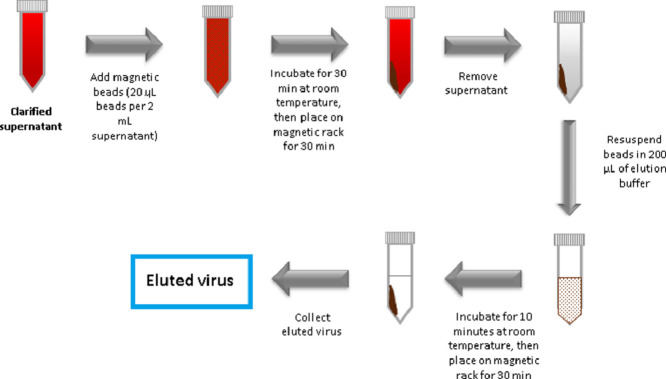

Specifications table

**Table:** 

Subject Area:	Immunology and Microbiology
More specific subject area:	Virology
Method name:	Magnetic capture, concentration, and conservation of flaviviruses and alphaviruses
Name and reference of original method:	Our method provides alternative applications for the Mag4C LV Kit protocol: https://www.ozbiosciences.com/index.php?controller=attachment&id_attachment=109, which is commercially available from OZ Biosciences.
Resource availability:	Kit: https://www.ozbiosciences.com/magnetic-virus-concentration/46-mag4c-lv-magnetic-lentivirus-capture.html Magnetic separation rack: https://www.ozbiosciences.com/magnetic-devices-for-magnetofection/72-magnetic-separation-rack.html

## Method details

### Background

**Fig. 1:**
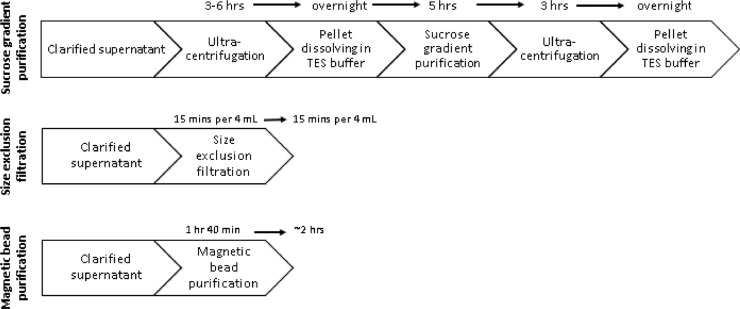
Process stages and length of time needed for sucrose gradient, size exclusion filtration, and magnetic bead purification. After supernatant containing virus is collected from infected multi-layered flasks, it is centrifuged and sterile filtered, producing clarified supernatant. This clarified supernatant is further purified and concentrated by sucrose gradient, size exclusion filtration, or magnetic bead purification. *Sucrose gradient*: Clarified supernatant is ultracentrifuged and virus is pelleted. After the pellet is dissolved overnight in tris-EDTA-saline (TES) buffer, a sucrose gradient ultracentrifugation is performed and the bands containing purified virus are collected. These bands are further concentrated with a final ultracentrifugation, and the resulting pellet is dissolved overnight in TES buffer. *Size exclusion filtration*: clarified supernatant is added in 4 mL increments to size exclusion filter tubes and centrifuged for 15 min. The total amount of time the filtration takes depends on the volume of clarified supernatant to be used. *Magnetic bead purification*: Magnetic beads are suspended in clarified supernatant and allowed to bind to the virus. The bead-virus complex is separated from the supernatant, and the virus is eluted from the beads and collected.

Virus production is an essential part of virology experiments and the quality of virus preparation (in terms of purification and yield) can affect the results of these experiments. Purified virus is critical for assays where contamination from the cell culture, e.g. cell culture medium additives, products of cell lysis (such as DNA, proteins, lipids, etc.), cytokines, and chemokines, may affect the results of the assays [1]. Purified virus is also needed for vaccine preparations [2]. The traditional process of gradient purification is time consuming, as it requires multiple steps (Fig. 1) with risk of contamination due to multiple handling steps and additionally, produces significant amounts of biohazards, including waste from the ultracentrifugation steps and excess gradient solution. Other methods for purification, such as size exclusion filtration, can still carry contaminants from the cell culture [3]. Thus, a more efficient method of effective virus purification is highly desirable.

Magnetic beads can be used for many biological applications, such as immunoprecipitation [4] and multiplexing [5]. They can also be utilized for virus capture and concentration [6]. However, commercial magnetic bead purification kits are mainly indicated for lentiviruses, retroviruses, and adenoviruses. In this study, we evaluated the efficacy of a commercial magnetic bead kit created for the concentration of lentiviruses and retroviruses, for use with an alphavirus (i.e. Mayaro virus (MAYV)) or a flavivirus (i.e. Zika virus (ZIKV)).

### Required reagents and equipment

• Minimal Eagle's Medium (MEM) (Corning, Corning, NY, USA)
• Newborn calf serum (NCS) (GE Healthcare, Chicago, IL, USA)
• Penicillin/streptomycin (Corning, Corning, NY, USA)
• 5-layer cell culture flasks (Corning, Corning, NY, USA)
• 50 mL conical tubes (Corning, Corning, NY, USA)
• 1.5 mL screw-cap tubes (Sarstedt, Nümbrecht, Germany)
• 15 mL conical tubes (Corning, Corning, NY, USA)
• 1 mL pipette, 200 µL pipette and tips (GeneMate, VWR, Radnor, PA, USA)
• Refrigerated centrifuge set to 4°C
• 0.22 micron vacuum filter/storage system (Corning, Corning, NY, USA)
• Mag4C LV kit (OZ Biosciences, San Diego, CA, USA)
  ○ Mag4C LV magnetic beads
  ○ Elution buffer
• Magnetic separation rack (OZ Biosciences, San Diego, CA, USA)
• Biosafety cabinet
• Incubator with 5% CO2 set to 37 °C
• 1X PBS

### Procedure

#### Cell cultivation and inoculation with virus

(1) Seed Vero cells in 5-layer cell culture flasks and grow to confluence in complete medium (MEM + 10% NCS + 1% P/S).
(2) Inoculate cells with MAYV or ZIKV and allow virus to multiply for 2 or 4 days, respectively.

#### Clarification of viral supernatant

(3) Decant supernatant from multi-layer flasks into 50 mL conical tubes.
(4) Centrifuge at 4 °C at 3500 rpm for 30 min.
(5) Vacuum filter (0.22 µm) the supernatants and store at 4 °C.

#### MAG4C LV virus capture and elution

(6) Add 2 mL of clarified supernatant to a 15 mL conical tube.
(7) Add 20 µL of Mag4C LV beads to the 2 mL of clarified supernatant.
(8) Incubate for 30 min at room temperature.
(9) Place tube on magnetic separation rack for 30 min.
(10) At this point, the beads should be held on the wall of the tube. Remove the supernatant and save as a sample (leftover clarified supernatant) at −80 °C.
(11) Remove the tube from the magnetic stand.
(12) Resuspend the beads in 200 µL of elution buffer.
  a At this point, the sample can be transferred to a smaller tube if desired.
(13) Incubate for 10 min at room temperature.
(14) Place tube on magnetic separation rack for 30 min.
(15) Collect the supernatant (this contains the eluted virus) and save as a sample (eluted virus) at -80 °C.

#### Optional: saving the magnetic beads

(16) Remove the tube from the magnetic stand.
(17) Resuspend the beads in 100 µL 1X PBS and store as a sample (leftover beads) at −80 °C.

## Method validation

Each sample collected during the magnetic bead purification process was subjected to protein estimation by bicinchoninic acid (BCA) protein assay, protein gel, western blot, and determination of the median tissue culture infectious dose (TCID50/mL) [6]. BCA results show that overall protein concentration decreases as more purification steps are performed (Table 1); this is likely due to the protein content from the lysed cells or protein present in the cell culture medium being removed with each step of the purification protocol.

**Table 1:** BCA results for ZIKV and MAYV samples. Protein concentrations in each sample were measured via BCA assay. As more purification steps are performed, the protein concentration decreases. This demonstrates how the magnetic beads are able to separate virus particles from other proteins in the clarified supernatant.

Sample	Protein Concentration			
	ZIKV (µg/µl)	Total protein	MAYV (µg/µl)	Total Protein
Clarified supernatant	6.14	12.28 mg/2 ml	5.82	291.00 mg/50 ml
Leftover clarified supernatant	6.56	13.12 mg/2 ml	5.08	254.00 mg/50 ml
Eluted virus	0.66	0.13 mg/0.2 ml	0.17	0.34 mg/2 ml
Leftover beads	0.06	0.01 mg/0.1 ml	0.03	0.02 mg/0.5 ml

The qualitative detection of total protein quantities (i.e., virus-specific proteins as well as protein impurities) was performed using protein gel analysis. 10 µg of protein per sample was separated on a 4–12% Bis-Tris gel (ThermoFisher Scientific, Waltham, MA, USA) under reducing conditions. The protein gel results correspond to the BCA results; specifically, as more purification steps are performed, less excess protein is observed on the gel, while the virus-specific proteins remain. It is also demonstrated that there are negligible amounts of protein remaining on the beads at the end of the magnetic bead purification process, indicating that the majority of virus has successfully been eluted.

The binding of virus to virus-specific monoclonal antibodies (specifically, ZIKV-specific antibody ZV54 and mouse anti-MAYV hyperimmune ascitic fluid) was evaluated by Western blot. Western blot analysis of the ZIKV samples using the ZIKV specific antibody ZV54 showed that the antibody was able to bind to the purified sample, indicating the presence of proteins characteristic of ZIKV. The intensity of the band decreases as more purification steps are performed, indicating possible loss of virus, which is consistent with the titer data (Table 2). Similarly, MAYV samples showed binding to mouse anti-MAYV hyperimmune ascitic fluid with a decrease in band intensity as further purification is performed.

**Table 2:** TCID50/mL values for samples collected during magnetic bead purification. The samples were serially diluted from 10E-1 to 10E-11 and used to inoculate Vero cells in 96-well plates. Each dilution had 8 replicates. After 2 and 5 days of incubation for MAYV and ZIKV, respectively, each well was evaluated for cytopathic effects (CPE) and marked positive or negative for CPE. TCID50/mL was calculated using the Reed and Muench method as described previously [8].

Sample	Titer (TCID50/mL)			
	ZIKV	Yield	MAYV	Yield
Clarified supernatant	1.08E + 04	2.16E + 04/2 mL	3.66E + 06	1.83E + 08/50 mL
Leftover clarified supernatant after removal of bead bound virus	6.56E + 03	1.31E + 04/2 mL	1.62E + 08	8.11E + 09/50 mL
Eluted virus	1.34E + 03	2.68E + 02 /200μL	2.64E + 08	5.27E + 08/ 2 mL
Leftover beads	5.00E + 01	5.00E + 00/100μL	1.32E + 07	6.60E + 06/500μL

Finally, TCID50/mL was measured in the ZIKV and MAYV samples in order to verify the viral titers [7]. The virus titer decreases as more purification steps are performed by about 10^–1^, indicating loss of virus particles during the purification process. The process was performed on small volumes for ZIKV (starting volume = 2 mL; Table 2) and large volumes for MAYV (starting volume = 50 mL; Table 2). The virus titer and yield after purification was higher for MAYV compared to ZIKV. M.

## Conclusion

In this study, we evaluated the efficiency and efficacy of using magnetic beads for the purification of two viruses, specifically ZIKV and MYAV. Characterization assays provided insight into the quantity of virus is in each sample as well as the purity of each sample. We also compared the quantity and purification with the time needed to generate each sample via different methods. Although sucrose gradient purification is the standard for purification in the field, it is time-consuming, taking 2–4 days to complete. Additionally, it is difficult to produce a highly concentrated, pure sample and also requires specific instrumentation including a gradient maker and fractionator. Size exclusion filtration can be performed quickly, but the quality of purification is very poor. The developed magnetic bead purification protocol can be used for the purification of ZIKV or MAYV (as well as other viruses in the respective groups) with minimum instrumentation need yet produces purified virus. However, the loss of virus with each purification step may limit the use of this approach where very high concentration and yield may be needed in downstream applications.

**Fig. 2:**
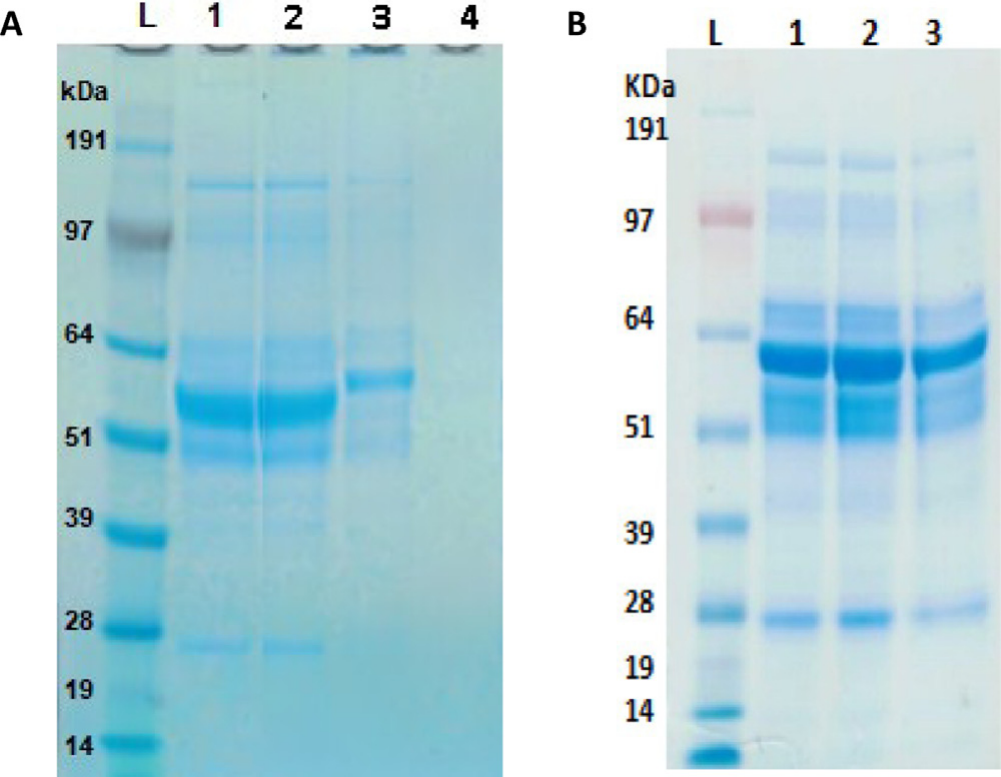
Protein gel results for samples collected during magnetic bead purification. Protein gel separation was performed on the samples under reducing conditions. The gels were stained with GelCode Blue Stain Reagent (ThermoFisher Scientific, Waltham, MA, USA). (A) Protein gel that shows the total protein in each sample for ZIKV, (B) protein gel that shows the total protein in each sample for MAYV. *L* = SeeBlue Plus2 Ladder (weights in kDa), 1 = clarified supernatant before purification, 2 = leftover clarified supernatant after removal of bead bound virus, 3 = eluted virus, 4 = leftover beads. In both MAYV and ZIKV samples, it is observed that the eluted virus lane has one concentrated band, as opposed to several dark bands in the clarified supernatants and leftover clarified supernatant. The leftover beads were included to show that most virus had been successfully eluted from the beads by the end of the protocol.

**Fig. 3:**
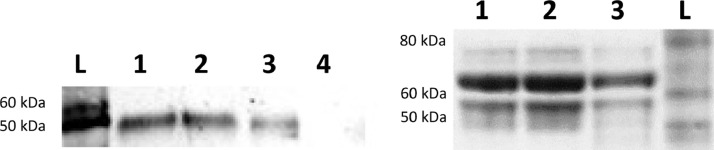
Western blot results for samples collected during magnetic bead purification. Protein separation was performed on the samples under reducing conditions and subsequently transferred onto a nitrocellulose membrane. The membrane was blotted with primary and secondary antibodies and chemiluminescence was detected. (A) Western blot for ZIKV samples using ZV54 as a primary antibody and HRP-conjugated goat anti-mouse as a secondary antibody in order to detect virus-specific proteins (B) Western blot for MAYV samples using mouse anti-MAYV hyperimmune ascitic fluid as a primary antibody and HRP-conjugated goat anti-mouse as a secondary antibody in order to detect virus-specific proteins. L = SeeBlue Plus2 Ladder (weights in kDa), 1 = clarified supernatant before purification, 2 = leftover clarified supernatant after removal of bead bound virus, 3 = eluted virus, 4 = leftover beads.

Magnetic bead purification successfully separates ZIKV and MYAV from various impurities and contaminants (Fig. 2), and the resulting virus preparation maintains its binding efficacy (Fig. 3) and infectivity (Table 2). In summary, this protocol is much more efficient than performing a sucrose gradient, as magnetic bead purification takes about 2 h to complete, while 2-4 days are needed to complete a sucrose gradient purification (Fig. 1).

## Disclaimer

Opinions expressed herewith are those of the author(s) and are not necessarily representative of those of the USUHS, DoD, or the United States Army, Navy, or Air Force.

## Appendix Supplementary materials

Image, application 1
