# Supplementary material for: Magnetic bead purification of enveloped alphavirus and flavivirus
Source: MethodsX. 2021 Oct 14;8:101549. doi: 10.1016/j.mex.2021.101549 (PMC8720801; doi:10.1016/j.mex.2021.101549)
Supplement: Supplementary file 1 [file mmc1.docx]

**Supplementary materials**


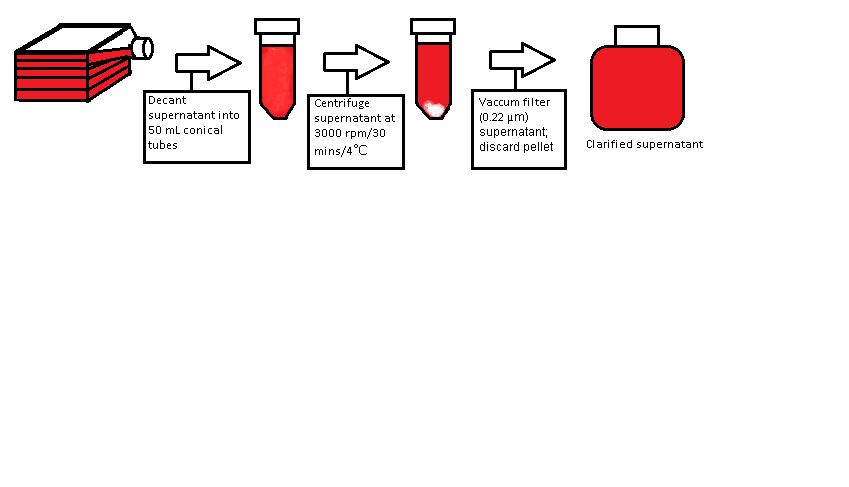


pellet

**Supplementary Figure 1. Process for clarifying virus supernatant.** Schematic of the processing of cell + virus supernatant into clarified supernatant.


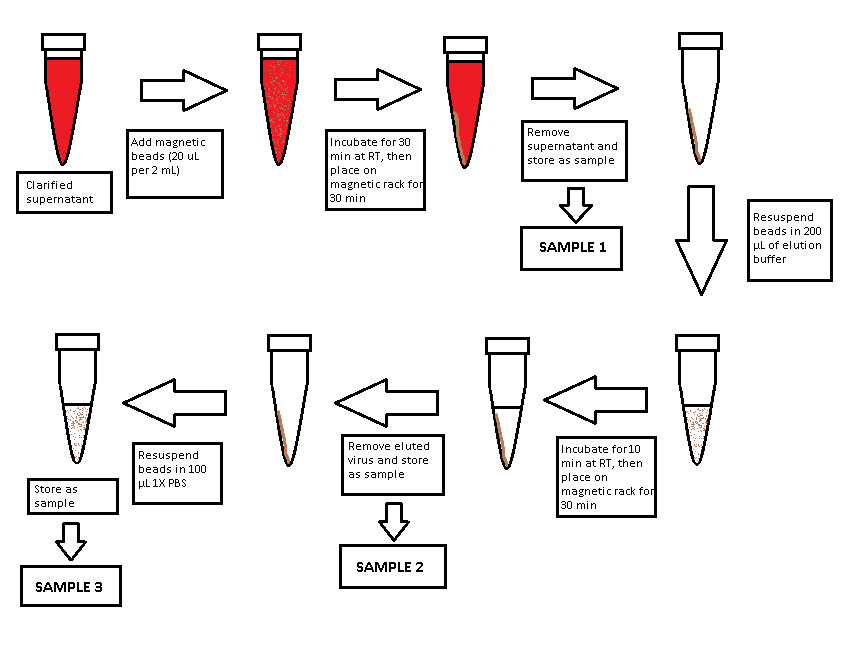
**Supplementary Figure 2. Schematic depicting the Mag4C LV bead purification process.** Visual schematic illustrating the process of Mag4C LV bead purification of virus and at which points samples are taken.

**Leftover beads**

**Eluted virus**

**Leftover supernatant**

**A**
